# Editorial: Insights in renal pharmacology: 2021

**DOI:** 10.3389/fphar.2022.1010691

**Published:** 2022-09-07

**Authors:** Norberto Perico, Matthew D. Griffin, Giuseppe Remuzzi

**Affiliations:** ^1^ Istituto di Ricerche Farmacologiche Mario Negri IRCCS, Bergamo, Italy; ^2^ Regenerative Medicine Institute (REMEDI) at CÚRAM Centre for Research in Medical Devices, School of Medicine, National University of Ireland Galway, Galway, Ireland

**Keywords:** chronic kidney disease, acute kidney disease, novel drugs, biologic agents, mechanistic targets

Kidney disease, either acute (AKI) or chronic (CKD), is an important contributor to morbidity and mortality from non-communicable diseases, and in under-resourced settings, also from communicable and maternal/neonatal diseases (Remuzzi and Horton, 2013; GBD Chronic Kidney Disease Collaboration, 2020). After the 1960s, the availability of renal replacement therapies (RRT) made possible the long-term application of life-saving but costly treatments for patients with end-stage kidney disease (ESKD) (Liyanage et al., 2015). In many countries, however, the lack of access to RRT means an unacceptable premature death for millions of children and adults (Liyanage et al., 2015). The global burden of CKD also extends well beyond the provision of RRT services. Indeed, acute and chronic loss of kidney function has been recognized as a risk factor for cardiovascular events independent of other conventional risk factors for cardiovascular disease (Sarnak et al., 2003; See et al., 2019), and as a risk multiplier for cardiovascular mortality in patients with hypertension and diabetes (Couser et al., 2011). There is evidence that slowing or halting kidney disease progression at early stages prevents the development of ESKD and cardiovascular complications, and can provide substantial economic benefits (Trivedi et al., 2002; KDIGO CKD Work Group, 2013). However, despite the available treatments and interventions, the global all-age burden of CKD has increased in the past three decades, highlighting the need for further research to dissect the in-depth pathophysiologic processes underlying kidney diseases and their progression to ESKD. Such mechanistic research is essential for the continued identification of targets for developing novel treatments for AKI and CKD and their consequences. The importance of this challenge has been further highlighted by the current COVID-19 pandemic, in which kidney involvement, through direct SARS-CoV-2 infection or as a consequence of multi-organ dysfunction, has emerged as a major risk factor for poor outcomes as well as one of the sequelae of long-COVID-19 (Perico et al., 2021a; Perico et al., 2021b).

Against this back-drop, this Research Topic provides a collection of original contributions that describe recent basic, pre-clinical, and clinical progress in defining the mechanisms and the efficacy of a diverse range of novel drugs, biologic agents and mechanistic targets with the potential to ultimately improve the management of AKI and CKD. Given the heterogeneity of the studies and the wide spectrum of disease-specific pathways and biological processes within the kidney, we briefly describe and discuss, in this Editorial, some of the overarching themes and clinical implications of the sixteen articles that comprise the Research Topic.

Tubular epithelial cells of the proximal segments of the nephron are particularly vulnerable to nephrotoxic and ischemic injuries that contribute to the development of AKI (Lombardi et al., 2016). Accumulating evidence indicates that kidney tubule epithelial cells are also key players in the development of renal fibrosis, the common end-point for all progressive kidney diseases leading to ESKD (Zoja et al., 2006; Boor et al., 2010). Four original articles from this collection have explored the mechanisms of action of novel drugs with respect to tubular cell protection. Tao et al. investigated the protective mechanisms of Dexmedetomidine (Dex), a highly selective α2-adrenoreceptor agonist, in *in vitro* cell damage and *in vitro*, in a mouse model of ischemia/reperfusion (I/R) injury—with a focus on ferroptosis, a non-apoptotic form of cell death that contributes to renal I/R injury (Zhang et al., 2021). It is characterized by mitochondrial shrinkage, increased mitochondrial membrane density, and lipid reactive oxygen species, as well as up-regulation of a unique set of genes including Acyl-CoA synthase long-chain family member 4 (ACSL4) (Li et al., 2019). As previously shown in other models, Dex administration mitigated tissue damage, inhibited ferroptosis, and downregulated the inflammatory response following renal I/R injury. The novelty of the study rests on the documentation, by elegant *in vitro* experiments, that the Dex-mediated protective effects occurred by inhibiting the expression and the activity of ACSL4 via α2-adenergic receptor. A second study (Long et al.) explores the effect and the mechanisms of a complementary therapy with a Chinese medicine formula on hypertension-induced renal injury. Treatment with the traditional medicine, Qingda granule (QDG), significantly attenuated hypertensive renal damage by partially preventing cellular apoptosis induced in renal tissues after angiotensin II (Ang II) infusion in mice, and by suppressing the p53 pathway. TUNEL staining documented that QDG treatment also markedly reduced Ang II-induced apoptosis in a renal tubular epithelial cell line. Besides being an example of a possible new therapeutic strategy for treating hypertensive renal injury, the study highlights the growing literature on the potential value of extracts from traditional Chinese medicines on kidney disease. Evidence suggests that senescence, a distinctive form of permanent cell cycle arrest of kidney tubular cells, influences kidney fibrosis (Schafer et al., 2018). By adjusting the culture conditions of their previously developed, conditionally immortalized proximal tubular epithelial cell line overexpressing the organic anion transporter 1 (ciPTEC-OAT1), Yang et al. documented the validity of this *in vitro* model to study kidney senescence. Indeed, culturing ciPTEC-OAT1 cells at 37°C induced a senescence phenotype characterized by increased expression of cell cycle arrest and anti-apoptosis markers, as well as of senescence-associated secretory phenotype factors. Notably, the model showed responsiveness to treatment with senolytic agents. These findings reveal a new avenue for pharmacological investigations of the sensitivity of senescence to novel drugs that selectively eliminate senescent cells by interfering with anti- and pro-survival signalling (Zhu et al., 2015). If validated *in vivo*, this targeted approach may represent an important new treatment option for preventing the development and progression of kidney fibrosis. Despite decades of calcineurin inhibitor use to modulate the immune system in transplantation and autoimmune diseases (Kamal and Doyle, 2022), the “off target” intracellular pathways underlying cyclosporine (CsA) nephrotoxicity remain ill defined. The study by Karolin et al. reports novel experimental results which suggest that, in kidney epithelial cells, CsA does not act via the calcineurin-NFAT (nuclear factor of activated T cells) axis, as in lymphoid cells, but through inhibition of p38 and PI3K/Akt kinases. It should be noted, however, that these findings do not exclude the possibility that calcineurin inhibitor nephrotoxicity also involves other renal cell molecular pathways.

Three other original articles published within the Research Topic focus on the mechanism(s) of podocyte injury and the potential for innovative agents to modulate this cell damage. Podocyte injury and related proteinuria are the most common features of glomerular disease, which is the leading cause of ESKD (Ruggenenti et al., 2012). The interest of research in this area is highlighted by the bibliometric analysis of Liu et al., who found that, in the last 30 years, global publications on podocyte injury have exponentially increased mainly, but not exclusively, in the field of diabetic kidney disease. Hot topics within this literature include autophagy, oxidative stress, inflammasome and microRNAs (miRNAs). These endogenous, small, single-stranded non-coding RNAs play important roles in many biological processes such as organogenesis, cell proliferation and apoptosis, typically by inhibiting the expression of their target genes at the protein level (Trionfini and Benigni, 2017). Relevant to this, the study by Wang et al. reports that, in a mouse remnant kidney model, overexpression of miRNA-671-5p aggravated podocyte injury, worsened kidney dysfunction and exacerbated renal fibrosis - effects that were limit by treating animals with oligonucleotides targeting miR-671-5p in a murine adriamycin nephropathy model. Thus, inhibiting miR-671-5p could potentially serve as a new approach to prevent podocyte injury and loss of renal function in proteinuric CKD. Other investigators contributing to the Research Topic (Yang et al.) report results that confirm, in a mouse model of diabetes, the beneficial therapeutic effects of SS31, a cell-permeable tetrapeptide that selectively targets the inner mitochondrial membrane as an antioxidant (Zhao et al., 2004). This study also provides some interesting new mechanistic insights in regard to the protective action of SS31 on podocytes in diabetic kidney disease.

Characterisation of novel pathways for CKD progression that offer insights for therapies is the subject matter of two other articles in the Research Topic. In their review, Curran and Koop expertly summarize the activities of aryl hydrocarbon receptor (AHR), a pleiotropic cell signalling molecule with diverse ligand-specific functions, which are implicated throughout the course of CKD. As the authors describe in detail, various organic endogenous molecules, in addition to exogenous drugs, chemicals, and dietary compounds, bind to and activate AHR to either promote glomerular and tubular damage or protect against kidney injury. These diverse responses are linked to AHR-induced crosstalk with transcription factors associated with kidney fibrosis, metabolism, and the renin-angiotensin system. This incisive review, of particular value to those with deeper interest in the molecular details of CKD pathophysiology, offers an understanding of the key regulatory role of AHR in physiological pathways that may lead to more targeted therapies for CKD. Similarly, in an extensive original study Xuan et al. delineate the pleiotropic effects of SGLT2 inhibitors, beyond the lowering of blood glucose levels and the control of glomerular hyperfiltration (Cortinovis et al., 2022). They show that the SGLT2 inhibitor, dapagliflozin, alleviates renal fibrosis by decreasing necroptosis/inflammation mediated by the receptor-interacting protein kinases 1 and 3 (RIP1 and RIP3) and the mixed-lineage kinase domain-like (MLKL) axis proteins (Choi et al., 2019) in a rat model of unilateral ureteral obstruction (UUO). Suppression of oxidative stress and apoptosis, along with improved mitochondrial function, are suggested to be mechanisms underlying the renoprotective properties of dapagliflozin. These interesting findings provide further rationale for the use of SGLT2 inhibitors to prevent non-diabetic CKD, which is the subject of ongoing clinical trials.

Two review articles address the emerging potential for and hurdles to the promotion of immune regulation as a strategy for modulating autoimmune diseases and kidney transplantation—a topic of growing interest within the biomedicine research community (Juneja et al., 2022). Moving from the observation that defects in interleukin-2 (IL-2) and T regulatory cells (Tregs) are known to contribute to systemic lupus erythematosus and lupus nephritis, Venkatadri et al. summarize the existing evidence for Treg-enhancement strategies involving IL-6, IL-2, IL-33, or a novel hybrid cytokine (termed IL233), that have been shown to induce remission in models of lupus nephritis. A number of alternative strategies for *in vivo* Treg activation and expansion for which encouraging *in vitro* and *in vivo* results have been reported are also described. These include a tumor necrosis factor receptor (TNFR)2-specific agonist, an agonistic OX40:Fc fusion protein, the selective inhibitor of Janus kinase 1 and 2, baricitinib, and mesenchymal stem cells (MSCs). Additionally, regulatory B cells (Bregs) have recently generated interest, having been identified as major drivers of tolerance in kidney transplantation and in autoimmune diseases (Dasgupta et al., 2020). Indeed, in both settings, impaired function and low numbers of Bregs have been reported—leading to a search for strategies to re-establish the lost homeostasis. Here, Garcia et al. discuss the relevance of *in vivo* and *in vitro* induction of Bregs and their potential use as therapeutic agents in kidney transplantation. One approach described by these authors is to boost natural Bregs through specific immunosuppressive agents such as belatacept or alemtuzumab, either alone or in combination with cell therapies (MSCs or Tregs) which have been shown in kidney transplant recipients to induce Bregs. More complex and debateable due difficulties in their identification and culture expansion, is the potential to use Bregs as an adoptive cellular therapy - although improved *in vitro* Breg induction systems are on the horizon.

With the transition from CKD to dialysis-dependent ESKD, comes a further significant increase in the risk for cardiovascular disease (CVD) compared with the general population. This accounts for over 50% of the mortality among dialysis patients (Sharma and Sarnak, 2017). The comprehensive review of Wang and Gao highlights the complex role of inflammatory cells and cytokines in the cross-talk between kidneys and heart in patients on chronic hemodialysis. The authors also propose potential pharmacological interventions that, by addressing key targets in the inflammatory cascade, could improve compliance with hemodialysis therapy and limit the risk of cardiovascular events. Along similar lines, the study by Tsai et al., based on real-world evidence, reports that dialysis patients treated with lipid-lowering agents display a marked reduction in the risk of mortality, hospitalization, and major adverse cardiovascular events. They suggest a clinical benefit of these drugs on major CV outcomes, challenging previous observational and randomized studies showing no advantage of this therapy in dialysis patients (Mach et al., 2020).

Finally, the last part of the collection includes three articles dealing with very disparate topics, but with some intriguing observations. The study by Deng et al. explores the clinical significance of antibody against the M-type phospholipase-A2-receptor (PLA2R), now a routine laboratory test for the characterization of patients with primary membranous nephropathy, as a guide to management. In a large group of patients with seropositive PLA2R-associated membranous nephropathy, they report that PLA2R antibody level above 150 RU/ml (actually a non-validated cut-off value) is associated with higher disease activity and worse prognosis, even under different immunosuppressive regimens. While these findings confirm results of previous studies on the predictive value of PLA2R antibody titre on disease outcome (Ruggenenti et al., 2017), they, unfortunately, include only the responses to older treatment regimens such as cyclophosphamide and calcineurin inhibitors and not the more recently introduced and better-tolerated B-cell depleting biologics (Ruggenenti et al., 2017). Tabibzadeh et al. focus on a still-debated issue in clinical practice and in the literature, namely the detection and risk of progression of early lithium-related nephrotoxicity. This cross-sectional cohort study of patients prescribed lithium salts for bipolar disorder, confirmed the independent negative effect of lithium exposure on kidney function, especially in patients with microcysts. Since early microcysts—detected only by magnetic resonance imaging (MRI) and not by CT scans or ultrasonography—might also reflect irreversible nephrotoxicity, it has been proposed that systematic MRI in this setting would help clinicians make difficult decisions in patients with a high suicidal risk at treatment discontinuation. The review article by Luo et al. highlights the effect of increased glomerular filtration rate on drug pharmacokinetics—the so-called “augmented renal clearance” (ARC) concept—with an emphasis on antibiotics. Sub-therapeutic exposure in patients with ARC, is an important reason of treatment failure. Thus, the authors examine the debate around the clinical identification of ARC, review the multiple potential mechanisms of this phenomenon, and propose approaches to ameliorate the efficacy of antibiotic treatment in the setting of ARC. Validation of the proposed approaches in future studies will be of high clinical significance.

To conclude, the collection of articles contributed to this Research Topic provides some excellent examples of recent advances which have deepened our knowledge of the complex mechanisms underlying acute and chronic renal tissue injury and explores target cellular pathways of novel potential pharmacologic and non-pharmacologic agents. These articles also serve to highlight that matching fundamental insights into the mechanisms of renal cell apoptosis, ferroptosis, senescence, inflammation and fibrosis to technological advances in the design of target therapies or to the repurposing of medicines with known disease-modulating effects, provides a strong template for the accelerated development of future treatments to slow or halt the progression of kidney diseases and prevent their complications.
